# The impact of cardiac loading on a novel metric of left ventricular diastolic function in healthy middle‐aged adults: Systolic–diastolic coupling

**DOI:** 10.14814/phy2.15129

**Published:** 2021-12-07

**Authors:** James P. MacNamara, Vivek Koshti, Katrin A. Dias, Erin Howden, Christopher M. Hearon, I‐Jou Cheng, Linda S. Hynan, Benjamin D. Levine, Satyam Sarma

**Affiliations:** ^1^ Institute for Exercise and Environmental Medicine Texas Health Presbyterian Hospital Dallas Texas USA; ^2^ University of Texas Southwestern Medical Center Dallas Texas USA; ^3^ Baker Heart and Diabetes Institute Melbourne Victoria Australia; ^4^ Tri‐Service General Hospital National Defense Medical Center Taipei City Taiwan; ^5^ Departments of Population & Data Sciences and Psychiatry University of Texas Southwestern Medical Center Dallas Texas USA

**Keywords:** coupling, diastole, echocardiography, hemodynamics

## Abstract

**Aims:**

Left ventricular (LV) restoring forces are primed by ventricular deformation during systole and contribute to cardiac relaxation and early diastolic suction. Systolic–diastolic coupling, the relationship between systolic contraction and diastolic recoil, is a novel marker of restoring forces, but the effect of left atrial pressure (LAP) is unknown. We tested preliminary methods of systolic–diastolic coupling comparing mitral annular velocities versus excursion distances and hypothesized a recoil/contraction distance ratio would remain unaffected across varying LAP, providing a surrogate for quantifying LV restoring forces.

**Methods and Results:**

Healthy subjects (*n* = 61, age 52 ± 5 years) underwent manipulation of LAP with lower body negative pressure (LBNP) and rapid normal saline (NS) infusion. Pulmonary capillary wedge pressure (PCWP; pulmonary artery catheter) and tissue Doppler imaging of the mitral annulus were measured. Two models of systolic–diastolic coupling––early diastolic excursion (ED_exc_)/systolic contraction (S_exc_) distances and e’/systolic (s’) velocities were compared. Velocity (e’/s’) coupling ratios varied significantly (mean e’/s’, slope = 0.022, *p* < 0.001) in relationship with PCWP (5–20 mmHg). Excursion (ED_exc_/S_exc_) coupling ratio did not vary in relationship with PCWP (ED_exc_/S_exc_: slope = −0.001, *p* = 0.19).

**Conclusions:**

Systolic–diastolic coupling using mitral annular distance ratios to standardize early diastolic recoil to systolic contraction was not significantly impacted by LAP, in contrast to coupling ratios using velocities. The pressure invariance of annular distance coupling ratios suggests this metric quantifies the efficiency of LV restoring forces by isolating systolic contributions to early diastolic restoring forces independent from changes in LAP.

## Graphical abstract

Left ventricular restoring forces are primed by ventricular deformation during systole and contribute to cardiac relaxation and early diastolic suction. Systolic–diastolic coupling, the relationship between systolic contraction and diastolic recoil, is a novel and load independent marker of restoring forces. The pressure invariance of the systolic–diastolic distance coupling ratios suggests this metric quantifies the efficiency of LV restoring forces by isolating systolic contributions to early diastolic restoring forces independent from changes in LAP.

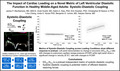

## 1 INTRODUCTION

Abnormalities in left ventricular (LV) relaxation are important criteria for diagnosing diastolic dysfunction. The most common clinical metric to characterize cardiac relaxation is e’, the early diastolic tissue Doppler velocity. e’ quantifies the velocity of early diastolic re‐lengthening, which is a complex and multifactorial process influenced by active relaxation mediated by sarco/endoplasmic reticulum Ca^2+−^ATPase (SERCA2a), left atrial‐left ventricular (LA‐LV) pressure gradient, and LV restoring forces (Nikolic et al., 1990; Opdahl et al., 2009; Shah et al., 2009; Smiseth et al., 1998). The isovolumetric decay of pressure in the LV (tau) and the E wave velocity can quantify the earliest phase of LV relaxation and the flow generated by the LA‐LV pressure gradient, respectively (Popovic et al., 2011; Prasad et al., 2007). By normalizing early diastolic recoil to the preceding systolic contraction, systolic–diastolic coupling represents the passive restoring forces recovered during early diastolic recoil. Systolic excursion (S_exc_) measures the longitudinal descent of the mitral annulus to the apex during systole and is often called mitral annular systolic planar excursion (MAPSE). Early diastolic excursion (ED_exc_) measures the distance the mitral annulus recoils during early diastole. The combination of ED_exc_ and late diastolic excursion, driven by the atrial contraction, would return the annulus to its presystolic position (Peverill, 2019). Our group has shown that systolic–diastolic coupling declines with age and is significantly reduced in heart failure with preserved ejection fraction (HFpEF; MacNamara et al., 2021).

As a marker of diastolic function, the hemodynamic influences of preload on systolic–diastolic coupling are important and unknown. In animal models, elastic recoil from restoring forces is responsible for generating diastolic suction and pressure decay within the ventricle even when the mitral valve is experimentally closed during diastole, blocking the LA pressure gradient (Nikolic et al., 1988). Additionally, long‐axis systolic excursion is closely correlated with early diastolic recoil in both human and animal studies suggesting LV deformation from systolic contraction primes the ventricle for early diastolic filling independent of left atrial pressures (Opdahl et al., 2009; Popovic et al., 2011). These animal studies demonstrate that restoring forces contribute to LV re‐lengthening independent of LAP and the LA‐LV pressure gradient. The purpose of this study was to determine if systolic–diastolic coupling was independent of LAP in healthy adults. We additionally sought to determine if a ratio of LV excursions or velocities better quantified pre‐load independent restoring forces. We hypothesized a diastolic recoil/systolic contraction ratio based on mitral annular distance would better estimate LV restoring forces by being unaffected across varying LAP.

## 2 METHODS

### 2.1 Study population

This study was an analysis of the baseline echocardiographic and hemodynamic data from a previously reported prospective study (Howden et al., 2018). Sixty‐one healthy adults aged 45–64 years were recruited. Exclusion criteria were hypertension, obesity (body mass index ≥30 kg/m^2^), untreated hypo‐ or hyperthyroidism, obstructive sleep apnea, chronic obstructive pulmonary disease, tobacco use during past 10 years, coronary artery disease, or structural heart disease. Subjects were sedentary and reported completing less than 90 min of exercise per week. All subjects were screened for abnormal systolic function and inducible cardiac ischemia with an exercise stress echocardiogram. Study procedures were approved by the institutional review boards of the University of Texas Southwestern Medical Center and Texas Health Presbyterian Hospital Dallas, and all subject signed informed consent. The study protocols conformed to the principles outlined in the Declaration of Helsinki.

### 2.2 Experimental protocol

All subjects underwent a cardiac unloading/loading protocol as previously described (Howden et al., 2018; Prasad et al., 2007). A Swan–Ganz catheter was placed using antecubital venous access and fluoroscopic guidance. The wedge position was confirmed by both fluoroscopy and change in pressure waveform. After 20 min of rest in the supine position, the subjects underwent hemodynamic measurements and an echocardiogram. Measurements included heart rate, beat by beat blood pressure, right atrial pressure, pulmonary capillary wedge pressure, and cardiac output by acetylene rebreathing (Jarvis et al., 1985). Cardiac pressures were obtained in triplicate during an end‐expiratory breath hold with care taken to avoid a Valsalva maneuver and were analyzed using an electronic data measurement system (BIOPAC Systems Inc.). Pulmonary capillary wedge pressure and right atrial pressures were obtained as mean pressure through the cardiac cycle and therefore represented all waveform components (Reddy et al., 2018).

Cardiac unloading was accomplished by a lower body negative pressure (LBNP) chamber and performed in two conditions, LBNP −15 mm Hg and LBNP −30 mm Hg. Subjects were given a 20‐min break and a light lunch after cardiac unloading. Baseline measurements were repeated, and cardiac loading was accomplished by rapid infusion (~200 ml/min) of warm isotonic normal saline (NS) and performed in two conditions, 15 ml/kg (NS +15) and 30 ml/kg (NS +30). Hemodynamic measurements and an echocardiogram were performed 5 min after the initiation of each level of LBNP and immediately after each dose of saline infusion.

### 2.3 Echocardiographic measurements

Echocardiograms were performed using an iE33 (Philips Medical System) at baseline and during each unloading and loading condition in the left lateral decubitus position. Mitral annular velocities were acquired using Tissue Doppler imaging and pulsed wave Doppler with a 5‐mm sample volume at tracking rate of 100 cm/s on the septal and lateral mitral annulus. Mitral inflow was acquired using pulsed wave Doppler with a 2‐mm sample volume at the mitral leaflet tips. Cardiac volumes were measured using 4‐beat 3D acquisition and were analyzed offline using QLab 9.0 (Philips). Doppler and 2D measures were analyzed offline using Xcelera (Philips).

Doppler velocities were measured in triplicate as the peak velocities of the entire systolic signal and early diastolic signal, taking care to avoid over or under gaining. Velocity time integrals were measured in triplicate by tracing the systolic signal to measure systolic excursion (S_exc_) and early diastolic signal to measure early diastolic excursion (ED_exc_). Care was taken to avoid the isovolumetric contraction and isovolumetric relaxation signal. Every systolic measurement was paired with an early diastolic measurement to prevent comparing different heart beats. Measures with a fused e’ and a’ signal were excluded. Lateral and septal signals were evaluated individually and averaged together resulting in a “mean” signal. Forty randomly chosen signals were repeated blinded to initial results by multiple readers (J.P.M., V.K.) for intra‐ and inter‐observer variability.

### 2.4 Systolic–diastolic coupling model

We modelled systolic–diastolic coupling as a ratio of early diastolic recoil/systolic excursion as previously described (MacNamara et al., 2021). Hooke's law (force = −K*distance) governs the compressibility of a spring, and the force needed to compress a spring scales linearly with distance of compression (Giuliodori et al., 2009). The efficiency of early diastolic recoil as a function of systolic contraction can be represented by dividing the ED_exc_ by the S_exc_, and the resulting ratio is the proposed metric of coupling (ED_exc_/S_exc_). Tissue Doppler imaging (TDI) was chosen to define LV longitudinal motion for several reasons. First, TDI permitted simultaneous measurement of the peak velocities and excursion distances of the mitral annulus for comparison (Clancy et al., 2017; Friedberg et al., 1985; Koh et al., 2010; Popovic et al., 2011). Second, TDI VTI is a previously validated measure of annular excursion (Hayashi et al., 2006; Manouras et al., 2009; Peverill, 2019). Third, TDI is less susceptible to poor image quality even at high levels of LBNP (Yuda et al., 2006). Finally, TDI provided the highest intra‐observer reproducibility and highest temporal resolution to define cardiac phases compared to other methods such as 3D or 2D imaging. The primary outcome of the study was the change in systolic and early diastolic LV longitudinal motion ratios, both excursion and velocity measures, as a function of left atrial pressures.

### 2.5 Statistical analysis

Statistical analysis was performed using SAS V9.4, and figures were made using GraphPad Prism 8.2.0 and SPSS Statistics 20. Continuous variables were represented as mean ± standard deviation. To assess the influence of PCWP on Doppler measures of cardiac motion, random regression coefficients models were used to assess changes in hemodynamic and cardiac parameters across all six loading conditions separately. The covariance between the intercept and slope for each model were tested and if this covariance was not significant, the results are presented for the model with the covariance set to 0. To test goodness of fit, mean *R*‐squared was calculated as the average of each individual linear regression, separate from the random regression coefficients model. Repeated measures one‐way ANOVA was used to assess changes in hemodynamic parameters across all six loading conditions. To compare excursion and velocity ratios across loading conditions, mixed effects model with one between (ratios) and one repeated factor (condition) with a compound symmetry model was used. A *p* < 0.05 was considered statistically significant.

## 3 RESULTS

Baseline characteristics of the population are represented in Table 1 as previously reported (Howden et al., 2018). Average age was 52 ± 5 years, and 52% identified as women. All subjects were normotensive at rest. Conventional echocardiographic parameters are shown in Table 1.

**TABLE 1:** Baseline demographics

Parameter	*n* = 61
Age (years)	52 ± 5
Gender (% women)	52%
Weight (kg)	75.1 ± 14.0
Height (cm)	170.1 ± 9.6
BMI	26.0 ± 3.2
SBP (mm Hg)	108.4 ± 8.5
DBP (mm Hg)	68.7 ± 6.5
MAP (mm Hg)	82.0 ± 6.6
LV end‐diastolic volume (ml)	94.6 ± 20.4
Peak E wave velocity	70.0 ± 15.2
Peak A wave velocity	51.5 ± 10.9
E/A	1.39 ± 0.3
Mean E/e’	7.4 ± 2.0

Note

Baseline demographics of cohort are represented as mean ± SD. Gender is represented at % female.

Abbreviations: BMI, body mass index; DBP, diastolic blood pressure; LV, left ventricle; MAP, mean arterial pressure; SBP, systolic blood pressure.

### 3.1 Effects of loading manipulation on cardiac hemodynamics

Comprehensive hemodynamic alterations with loading and unloading are shown in Table 2. By design, pulmonary capillary wedge pressure was significantly altered by both unloading and loading from LBNP and rapid NS infusion (5.2 ± 1.4 to 19.6 ± 1.8 mmHg, *p* < 0.001 across conditions). There was an increase in systolic blood pressure (103 ± 10 to 114 ± 13 mmHg, *p* < 0.001 across conditions) and a decrease in diastolic blood pressure (range: 70 ± 8 to 67 ± 7 mmHg, *p* < 0.001 across conditions). Heart rate increased both with loading and unloading (*p* < 0.001 across conditions). Cardiac output increased from LBNP to NS infusion (3.56 ± 0.81 to 7.12 ± 1.67 L/min, *p* < 0.001 across conditions).

**TABLE 2:** Cardiac hemodynamics across loading conditions

Parameter	LBNP −30 mm Hg	LBNP −15 mm Hg	Baseline LBNP	Baseline saline	Saline +15 ml/kg/min	Saline +30 ml/kg/min	*p* across conditions
Pulmonary capillary wedge pressure (mm Hg)	5.2 ± 1.4^a^	7.3 ± 1.6^a^	11.8 ± 1.7	10.5 ± 1.7^a^	16.1 ± 1.9^a^	19.6 ± 1.82^a^	<0.001
Right atrial pressure (mm Hg)	3.6 ± 1.3^a^	5.0 ± 1.3^a^	8.7 ± 1.4^a^	7.2 ± 1.4^a^	11.3 ± 1.4^a^	14.2 ± 1.7^a^	<0.001
Systolic blood pressure (mm Hg)	103 ± 10^a^	107 ± 9	108 ± 9	108 ± 9	110 ± 11	114 ± 13^a^	<0.001
Diastolic blood pressure (mm Hg)	70 ± 8^a^	68 ± 7^a^	69 ± 7	64 ± 6^a^	65 ± 8	67 ± 7^a^	<0.001
Mean arterial pressure (mm Hg)	81 ± 8	81 ± 7	82 ± 7	79 ± 6^a^	80 ± 7	83 ± 8	<0.001
Heart rate (bpm)	73 ± 11^a^	67 ± 9	66 ± 8	72 ± 9^a^	80 ± 10^a^	82 ± 11^a^	<0.001
Cardiac output (L/min)	3.56 ± 0.81^a^	4.24 ± 0.85^a^	4.78 ± 0.79	5.28 ± 0.96^a^	6.94 ± 1.61^a^	7.12 ± 1.67^a^	<0.001
Stroke volume (ml)	50.3 ± 15.9^a^	65.4 ± 18.2^a^	74.0 ± 15.3	74.7 ± 16.0	87.1 ± 19.4^a^	87.6 ± 18.9^a^	<0.001

Note

The impact of loading and unloading on cardiac hemodynamics including intra‐cardiac pressures, blood pressure, heart rate, cardiac output (by acetylene rebreathe), and stroke volume. Values are represented as mean ± SD. ANOVAs are presented as *p* across conditions.

^a^ Statistically different from baseline LBNP value (Tukey correction for multiple comparisons; *n* = 61, 52% identified as women, 48% identified as men).

### 3.2 Effects of loading manipulation on individual metrics of longitudinal annular motion

**FIGURE 1:**
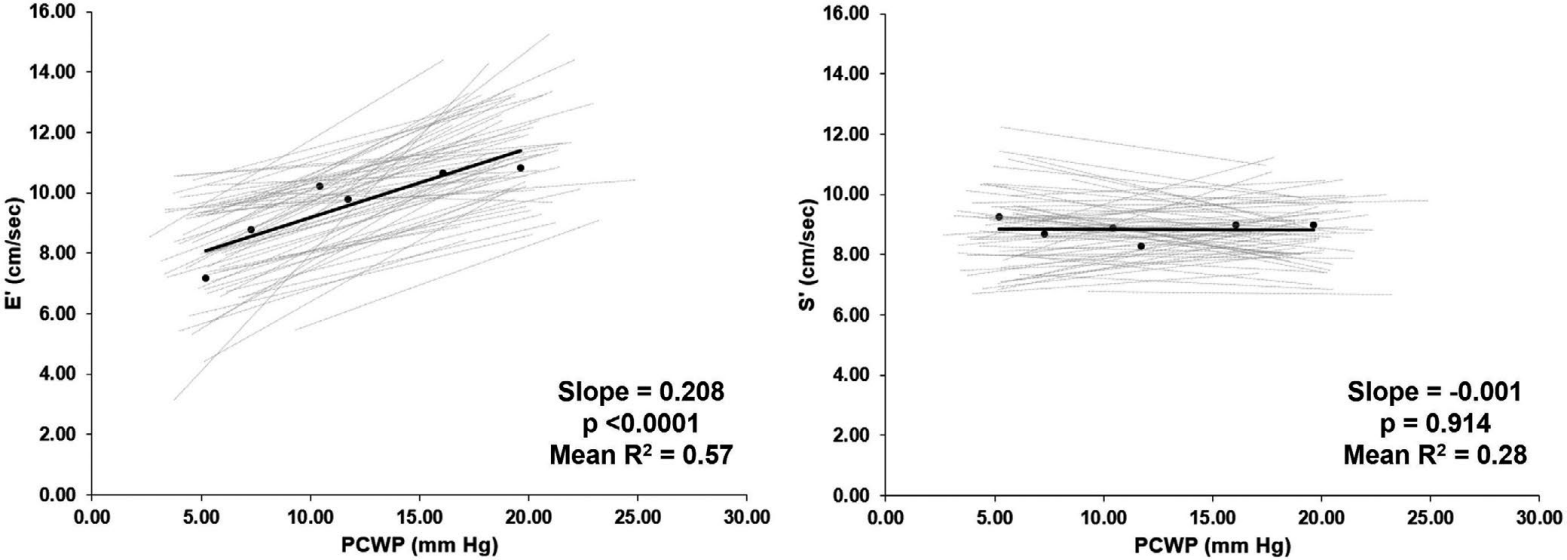
Early diastolic velocity (E’) and systolic velocity (S’) have differential response to preload. Average (black) and individual linear regressions (gray) for subjects’ e’ (left) and s’ (right) as influenced by pulmonary capillary wedge pressure (PCWP). The slope value shown is the fixed effect slope and *p* value of a random regression model. Slope and *p* value shown derived from random regression coefficients model. Mean *R* ^2^ is derived from linear regressions of individual participants (*n* = 61, 52% identified as women, 48% identified as men)

**FIGURE 2:**
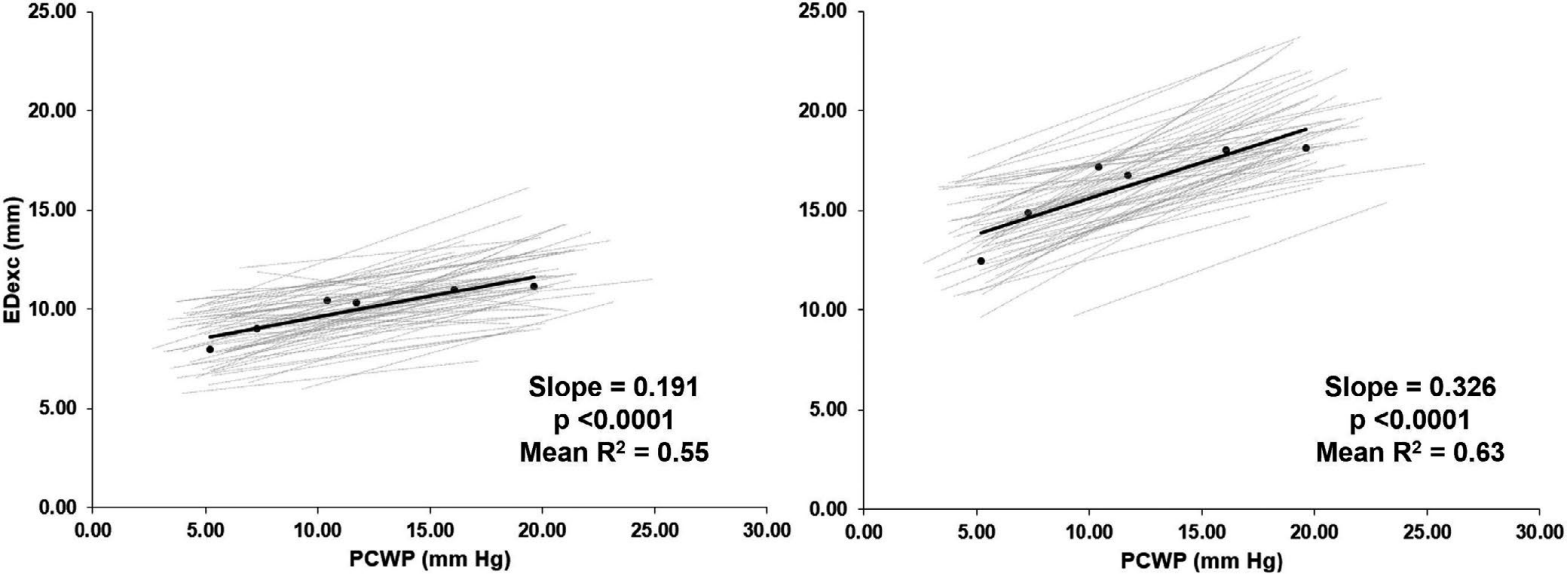
Early diastolic excursion (ED_exc_) and systolic excursion (S_exc_) have similar response to preload. Average (black) and individual linear regressions (gray) for subjects’ ED_exc_ (left) and S_exc_ (right) as influenced by pulmonary capillary wedge pressure (PCWP). The slope value shown is the fixed effect slope and *p* value of a random regression model. Slope and *p* value shown derived from random regression coefficients model. Mean *R* ^2^ is derived from linear regressions of individual participants (*n* = 61, 52% identified as women, 48% identified as men)

Changes in annular velocities and excursion distances are shown in Table 3, Figures 1 and 2. Early diastolic velocities changed significantly in response to alterations in PCWP (e’; fixed effects slope = 0.208; *p* < 0.001, mean *r* ^2^ = 0.57). There was no significant effect of changes in PCWP on systolic velocities (s’; fixed effects slope = −0.001, *p* = 0.914, mean *r* ^2^ = 0.28). e’ increased by 51.3% from LBNP −30 mm Hg to NS +30 ml/kg/min. s’ decreased by 4.5% from LBNP −30 mm Hg to NS +30 ml/kg/min. Both systolic and early diastolic excursion distances were significantly changed as PCWP was altered (S_exc_: fixed effects slope = 0.326, *p* < 0.001, mean *r* ^2^ = 0.55 and ED_exc_: fixed effects slope = 0.191, *p* < 0.001, mean *r* ^2^ = 0.63). S_exc_ increased by 45.6% from LBNP −30 mm Hg to NS +30 ml/kg/min. ED_exc_ increased by 40.7% from LBNP −30 mm Hg to NS +30 ml/kg/min (Figure 2).

**TABLE 3:** Individual metrics of longitudinal motion across loading conditions

Parameter	LBNP −30 mm Hg	LBNP −15 mm Hg	Baseline LBNP	Baseline Saline	Saline +15 ml/kg/min	Saline +30 ml/kg/min	Δ LBNP –30 to NS +30	*p*
ED_exc_/S_exc_	0.64 ± 0.10	0.61 ± 0.08	0.62 ± 0.06	0.61 ± 0.06	0.61 ± 0.06	0.62 ± 0.06	−0.03 ± 0.10 (−4.1%)	0.19
ED_exc_ (mm)	7.94 ± 1.62	9.04 ± 1.55	10.31 ± 1.42	10.42 ± 1.71	10.99 ± 1.66	11.16 ± 1.63	3.23 ± 1.81 (40.7%)	<0.0001
S_exc_ (mm)	12.44 ± 2.20	14.88 ± 2.13	16.76 ± 1.90	17.18 ± 2.06	18.04 ± 1.96	18.12 ± 2.00	5.68 ± 2.25 (45.6%)	<0.0001
E’/S’	0.79 ± 0.16	0.96 ± 0.19	1.13 ± 0.19	1.09 ± 0.22	1.16 ± 0.20	1.16 ± 0.19	0.42 ± 0.20 (53.8%)	<0.0001
E’ (cm/s)	5.96 ± 0.97	7.38 ± 1.46	8.35 ± 1.48	8.88 ± 1.61	9.57 ± 1.46	9.85 ± 1.75	3.68 ± 2.07 (51.3%)	<0.0001
S’ (cm/s)	7.74 ± 1.23	7.78 ± 1.10	7.45 ± 1.01	8.27 ± 1.16	8.33 ± 1.14	8.49 ± 1.02	−0.42 ± 1.87 (−4.5%)	0.91

Note

The impact of loading and unloading on metrics of longitudinal ventricular motion. Values are represented as mean ± SD and are mean values of both the septal and lateral annulus. Fixed effects are presented as *p* of variable associated with PCWP (*n* = 61, 52% identified as women, 48% identified as men). e’ = early diastolic velocity, s’ = systolic velocity, ED_exc_ = early diastolic excursion, S_exc_ = systolic excursion.

### 3.3 Effects of loading manipulation on metrics of systolic–diastolic coupling

**FIGURE 3:**
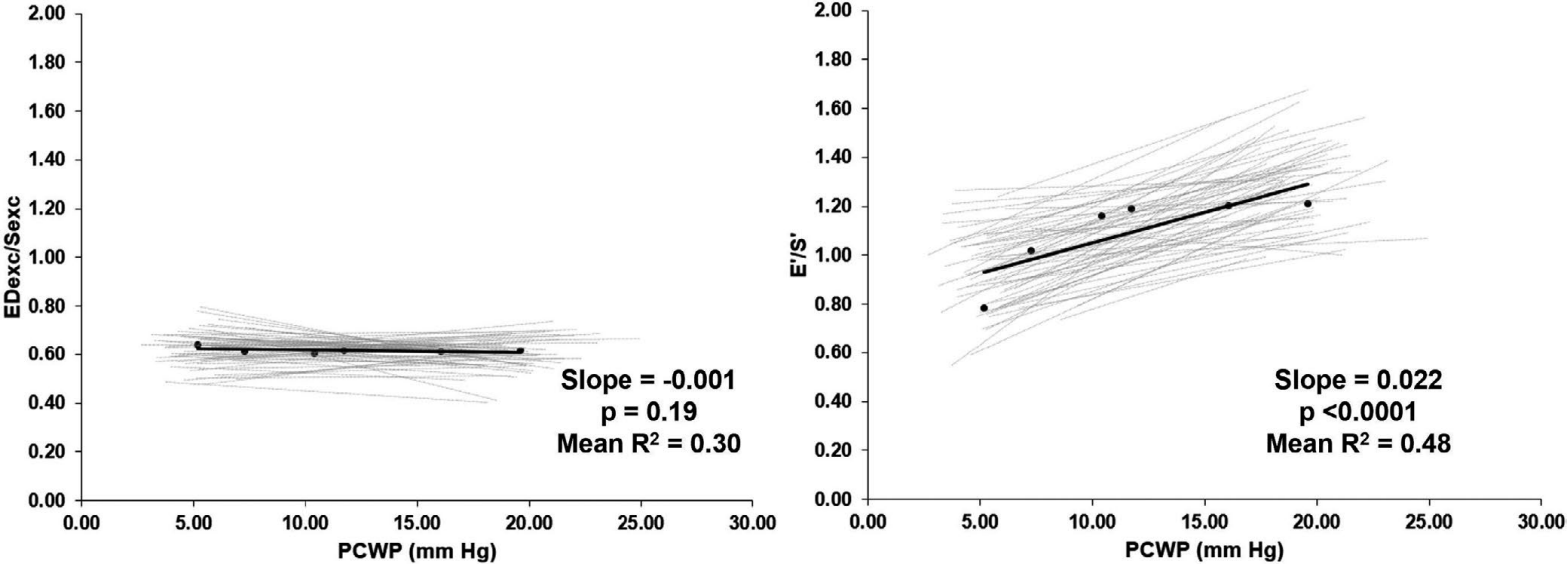
Metrics of systolic–diastolic coupling across loading conditions show different response to preload: left panel demonstrates no change in excursion (ED_exc_/S_exc_) ratio with changes to pulmonary capillary wedge pressure (PCWP) while right panel show a significant change in velocity (E’/S’) ratio with changes to PCWP. Individual linear regressions for subjects are shown in gray and average regression shown in black. The slope value shown is the fixed effect slope and *p* value of a random regression model. Mean *R* ^2^ is derived from linear regressions of individual participants (*n* = 61, 52% identified as women, 48% identified as men)

Excursion ratios are shown in Table 3 and Figure 3. The baseline mean excursion ratio was 0.62 ± 0.06. The mean ED_exc_/S_exc_ ratio did not significantly change with PCWP (fixed effects slope = −0.001, *p* = 0.19, mean *r* ^2^ = 0.30), and the results were unchanged when adjusted for gender (adjusted *p* = 0.51 for gender, adjusted *p* = 0.17 for PCWP). The ED_exc_/S_exc_ ratio decreased by 4.1% from LBNP −30 mm Hg to NS +30 ml/kg/min (Figure 2). In contrast, the e’/s’ velocity ratio changed significantly with PCWP (fixed effects slope = 0.022, *p* < 0.0001, mean *r* ^2^ = 0.63). The mean e’/s’ ratio increased 53.8% from LBNP −30 mm Hg to NS +30 ml/kg/min. This relative change of the annular velocity ratio was significantly different compared to the annular excursion ratio as a function of loading condition (interaction < 0.001).

### 3.4 Intra‐subject variability and reproducibility of excursion metrics

Of 732 septal and lateral ED_exc_/S_exc_ measurements, only 10 (1.4%) could not be performed due to fusion of e’ and a’ or image quality. Mean coefficient of variation for each intra‐subject excursion ratio at baseline was 6.0% for septal annulus and 5.3% for lateral annulus. Typical error for between blinded interpreters was 3.5% for S_exc_, 5.1% for ED_exc_, and 5.1% for ED_exc_/S_exc_ ratio. Intra‐observer typical error was 4.4% for ED_exc_/S_exc_ ratio.

## 4 DISCUSSION

The key finding of this study is that systolic–diastolic coupling using mitral annular distance ratios to standardize early diastolic recoil to systolic contraction was not significantly impacted by changes in LAP, in contrast to coupling ratios using annular velocities. The pressure invariance of annular distance coupling ratios suggests this metric estimates the efficiency of LV restoring forces by linking systolic contributions to early diastolic restoring forces independent from changes in LAP. By normalizing the LV early diastolic recoil excursion to the systolic excursion, we were able to further characterize this novel measure of diastolic function.

Assessment of diastolic function is complex and represents the summed components of LA‐LV pressure gradients, ventricular restoring forces, and active unwinding of actin‐myosin cross‐bridges facilitated by removal of cytosolic calcium via SERCA2a activity (Bers, 2002). Clinically reportable measures of diastolic function, including E wave velocity, E/A ratio, and e’ velocity, predominantly reflect the LA‐LV pressure gradient. These metrics are naturally load dependent as venous return influences this gradient (Choong et al., 1987, 1988; Prasad et al., 2007). The isovolumetric decay of pressure in the LV (tau) can measure the earliest phases of LV relaxation but is not synonymous with early diastolic LV lengthening and occurs predominantly prior to LV filling (Popovic et al., 2011; Scalia et al., 1997). Finally, restoring forces from the systolic contraction have an independent influence on LV lengthening (Nikolic et al., 1988). While our metric systolic–diastolic coupling is unlikely to directly measure restoring forces alone, we theorize that the ED_exc_/S_exc_ ratio measures efficiency of diastolic excursion as a function of restoring forces. This data supports our previous study which found that the ED_exc_/S_exc_ ratio declined with age and was further decreased in patients with HFpEF (MacNamara et al., 2021). Further study in clinical cohorts are needed to evaluate the clinical utility of this method in patients other cardiomyopathies and may be a particular interest in cardiac amyloidosis or hypertrophic cardiomyopathy.

### 4.1 Spring model of systolic–diastolic coupling

The principle of systolic–diastolic coupling describes the relationship between energy stored in the myocardium during systole, likely via compression of titin and the extracellular matrix, and then recovered during early diastolic recoil (Choong et al., 1988; Granzier & Labeit, 2002; Prasad et al., 2007). In animal and human studies, changes in systolic contraction have been associated with alterations in diastolic filling, as assessed by intraventricular pressure gradients, ventricular filling, and left ventricular lengthening rate (Firstenberg et al., 2008; Ohte et al., 2001; Winter et al., 2004). While the relationship between systole and diastole has been described, the exact nature of this relationship and how it can be quantified is unknown (Koh et al., 2010; MacNamara et al., 2021; Yip et al., 2002). In this study, we further characterize the systolic–diastolic coupling relationship: as systolic contraction was lessened or augmented across loading conditions, diastolic recoil similarly changed. This relationship may be due to compressive force storing potential energy in the myocardium that is released during early diastole and was independent of heart size and left atrial pressure.

### 4.2 Prior models of systolic–diastolic coupling

While systolic–diastolic coupling has not been measured across measured differences in LAP, Notomi et al. evaluated the relationship with systolic twist, diastolic untwisting, and intraventricular pressure gradients (Notomi et al., 2006). Enhanced LV twist during exercise was associated with enhanced LV untwisting and a high IVPG, which suggested the relationship between systolic twisting and diastolic untwisting was maintained. Increased venous return from activation of the muscle pump results in an increase in LAP during exercise, and thus it can be hypothesized that systolic twisting and diastolic untwisting remained linked as LAP increased (Wolsk et al., 2017). The present study further this concept by demonstrating the parallel enhancement of systolic contraction and early diastolic recoil in the longitudinal plane and shows unchanged systolic–diastolic relationship with isolated preload manipulation. How changes in afterload and cardiac contractility, which occur during exercise, individually effect systolic–diastolic coupling remains unknown.

Prior studies have shown a linear relationship between systolic contraction and early diastolic recoil in healthy adults and have found heterogeneous relationships in disease states, such as heart failure, dilated cardiomyopathy, and sepsis (Clancy et al., 2017; Friedberg et al., 1985; Koh et al., 2010). Friedberg et al. found a linear relationship between e’ and s’ in children and the s’/e’ ratio was altered in children with dilated cardiomyopathy while Koh et al. found s’ and e’ remained associated in children with LV non‐compaction (Friedberg et al., 1985; Koh et al., 2010). Clancy et al. found diminished e’/s’ ratio in adults with severe sepsis and a strong association with “diastolic dysfunction” (Clancy et al., 2017). These studies are cross‐sectional and use velocities as their metric of systolic–diastolic coupling, which may account for the heterogeneous results in patients with varied loading conditions (HF vs. sepsis, for instance). Other models have evaluated end‐systolic length or short axis diameter, but these do not necessarily account for the amount of shortening during systole (Zile et al., 1989). The systolic excursion distance and the early diastolic recoil distance are tightly linked, but the evaluation of this relationship has only been explored in a few cross‐sectional studies (Peverill, 2019; Yip et al., 2002). Yip et al. found a close linear correlation between systolic excursion and early diastolic recoil, measured by M‐mode and a nonlinear correlation between s’ and e’ velocities (Yip et al., 2002). The present study demonstrates that mitral annular excursions are tightly linked across loading conditions and compared to mitral annular velocities, provide a more robust quantification of efficient restoring forces in healthy adults.

This study demonstrated that e’ significantly decreased proportionally with reductions in PCWP while s’ increased nominally, highlighting the contrasting effects of loading on annular velocities. As a result, the e’/s’ velocity ratio was significantly altered by changes in left atrial pressure. To put this disassociation into clinical context, the velocity ratio diverges at levels of cardiac unloading similar to the seated position (LBNP −15 mmHg) and standing (LBNP −30 mmHg; Cooke et al., 1985; Goswami et al., 2019). In contrast, the ED_exc_ and S_exc_ decreased in parallel with unloading and increased in parallel with loading likely reflecting changes in ventricular volume as a function of filling pressure. With both systolic and diastolic excursion distances changing in parallel, the coupling ratio remained constant and was unaffected by changes in PCWP. We observed in this middle aged, otherwise healthy cohort that approximately 62% of the systolic excursion was recovered during early diastole, independent of LA pressure. A one‐to‐one coupling between systole and early diastole would not be expected, as the atrial contraction contributes to completing LV filling and re‐lengthening during late diastole. A decrease in the ED_exc_/S_exc_ ratio could suggests inefficient ventricular restorative forces after systolic excursion, potentially caused by abnormalities in the myocardial tissue, titin or the extracellular matrix, which are frequently seen in diseases such as HFpEF (Heerebeek et al., 2012; Kruger & Linke, 2009). Furthermore, a very high ratio, approaching 1, could signal an advanced cardiomyopathy with reduced systolic excursion and little contribution of atrial contraction to re‐lengthening.

### 4.3 Clinical implications

In a recent study, our group demonstrated that ED_exc_ and S_exc_ are tightly linked to cardiovascular changes of aging in healthy adults (MacNamara et al., 2021). The ED_exc_/S_exc_ ratio decreases by 15% per decade of life along with other markers of diastolic function (Tau, E/e’, E/A, and e’). The ED_exc_/S_exc_ ratio was superior for discriminating HFpEF from healthy age‐matched controls to E/e’ or e’. Diastolic function changes with healthy aging, particularly sedentary aging, and can confound the echocardiographic assessment of diastolic dysfunction (Prasad et al., 2007; Shah et al., 2017). Markers that decline with age and are significantly influenced by the LA‐LV pressure gradient may not adequately differentiate healthy aging and HFpEF. By removing the influence of the LA‐LV pressure gradient, the ED_exc_/S_exc_ ratio was able to quantify inefficient restoring forces present in HFpEF, while e’ was not. In the prior study, ED_exc_/S_exc_ ratio was modestly associated with resting LAP in HFpEF but not controls. While it is possible that systolic–diastolic coupling changes as HFpEF develops to become sensitive to preload, the present study suggests an alternative hypothesis: both a low ED_exc_/S_exc_ ratio and increased resting LAP are markers of advanced diastolic dysfunction. Like systolic–diastolic coupling in HFpEF, the relationship between systolic twisting and diastolic twisting was blunted in hypertrophic cardiomyopathy patients in Notomi et al.'s study (Notomi et al., 2006). Further studies are warranted to determine the prognostic capabilities of the ED_exc_/S_exc_ ratio in other cardiomyopathies.

No single marker of left ventricular diastolic function exists, and current clinical guidelines use multiple markers to identify “diastolic dysfunction” (Nagueh et al., 2016). These markers are either surrogates for left atrial pressure (increased E/e’, left atrial volume index, and right ventricular systolic pressure) or are directly influenced by the LA‐LV pressure gradient (mitral inflow, e’ velocities; Choong et al., 1987, 1988; Nagueh et al., 2001; Prasad et al., 2007). By definition, these markers are significantly influenced by changes in left atrial pressure and when combined are effective at detecting chronic elevations in left atrial pressure (Andersen et al., 2017). Yet, focusing the definition of “diastolic dysfunction” as increases in LA pressure risks missing more subtle abnormalities in ventricular function that could lead to earlier identification of disease, and perhaps initiation of preventative efforts (Borlaug et al., 2010; Kosmala & Marwick, 2020). By normalizing diastolic recoil to systolic excursion, systolic–diastolic coupling has the potential to quantify the efficiency of restoring forces involved in lengthening and provide a novel marker of diastolic function that is independent of the left atrial pressure. A lower excursion ratio would suggest that less recoil is obtained by release of compressive forces and other energetic processes must restore ventricular length (MacNamara et al., 2021). While this has been shown in a small cohort of HFpEF patients, further studies will be needed to determine systolic–diastolic coupling's clinical role with cardiovascular disease with and without evident systolic dysfunction.

### 4.4 Strengths and limitations

This study included a careful manipulation of cardiac preload. PCWP was measured at end‐expiration and measured through the waveform to estimate mean left atrial pressure (Reddy et al., 2018). No subjects had evidence of pulmonary venous stenosis or cardiac abnormality that would compromise the use of PCWP as a surrogate for LAP. The echocardiographic measurements were made immediately following the pressure measurements during a steady state loading condition, permitting direct comparison. This study has several limitations. First, systolic–diastolic coupling has no gold standard for comparison. This study evaluated the impact of cardiac loading on systolic–diastolic coupling on a healthy middle‐aged cohort as assessed by the excursion ratio to provide a “normal” value and physiologic understanding for future study of pathologic effects. Second, we are unable to assess the isolated effects of cardiac preload without corresponding changes in blood pressure and heart rate that may modulate the relationship between systolic contraction and early diastolic recoil, though these changes were small across very large changes in LA pressure. Third, the tissue Doppler measurements only account for the longitudinal motion of the heart and neglect both radial, circumferential and twisting motion. However, previous studies have demonstrated longitudinal motion is the primary determinant of stroke volume (Carlsson et al., 2007). Moreover, longitudinal metrics, particularly tissue Doppler, are more available and reproducible than circumferential metrics. We propose the excursion ratio as a metric of efficiency and not a single metric of diastolic function, which is a complex and multifactorial process of cardiac filling and atrial function. Fourth, this data does not support conclusions about the effects of isolated afterload changes or the presence of systolic dysfunction on systolic–diastolic coupling. It remains unknown if systolic–diastolic coupling remains preload independent in pathologic conditions with an altered end‐systolic pressure or end‐diastolic pressure volume relationship. Future studies are needed to determine how alterations of systolic function other than preload influence systolic–diastolic coupling.

## 5 CONCLUSION

Early diastolic re‐lengthening is significantly influenced by prior systolic excursion. A ratio of these measures, the ED_exc_/S_exc_, is a metric of systolic–diastolic coupling and a potential novel metric of efficient diastolic restoring forces. In contrast to measures of velocity (e’ and e’/s’) this relationship appears to isolate left ventricular restoring forces independent from LA pressure. This novel metric may be useful in identifying subclinical diastolic dysfunction.

## CONFLICT OF INTEREST

None declared.

## AUTHOR CONTRIBUTIONS

Conception and Design of Work: James P. MacNamara, Katrin A. Dias, Christopher M. Hearon, Linda S. Hynan, Benjamin D. Levine, and Satyam Sarma. Acquisition and Analysis: James P. MacNamara, V.K., E.H., I.C., L.H, Benjamin D. Levine, and Satyam Sarma. Drafting and Revising Intellectual Content: James P. MacNamara, V.K., Katrin A. Dias, Christopher M. Hearon, E.H., I.C., Linda S. Hynan, Benjamin D. Levine, and Satyam Sarma. All authors approved the final version of the manuscript, agree to be accountable for all aspects of the work in ensuring that questions related to the accuracy or integrity of any part of the work are appropriately investigated and resolved, and all persons designated as authors qualify for authorship, and all those who qualify for authorship are listed.
